# Lipids in hepatic glycogen storage diseases: pathophysiology, monitoring of dietary management and future directions

**DOI:** 10.1007/s10545-015-9811-2

**Published:** 2015-01-30

**Authors:** Terry G. J. Derks, Margreet van Rijn

**Affiliations:** Section of Metabolic Diseases, Beatrix Children’s Hospital, University of Groningen, University Medical Center Groningen, PO Box 30 001, 9700 RB Groningen, The Netherlands

## Abstract

Hepatic glycogen storage diseases (GSD) underscore the intimate relationship between carbohydrate and lipid metabolism. The hyperlipidemias in hepatic GSD reflect perturbed intracellular metabolism, providing biomarkers in blood to monitor dietary management. In different types of GSD, hyperlipidemias are of a different origin. Hypertriglyceridemia is most prominent in GSD type Ia and associated with long-term outcome morbidity, like pancreatitis and hepatic adenomas. In the ketotic subtypes of GSD, hypertriglyceridemia reflects the age-dependent fasting intolerance, secondary lipolysis and increased mitochondrial fatty acid oxidation. The role of high protein diets is established for ketotic types of GSD, but non-traditional dietary interventions (like medium-chain triglycerides and the ketogenic diet) in hepatic GSD are still controversial and necessitate further studies. Patients with these rare inherited disorders of carbohydrate metabolism meet several criteria of the metabolic syndrome, therefore close monitoring for cardiovascular diseases in ageing GSD patients may be justified.

## Introduction

To date there are 14 types of glycogen storage diseases (GSD), which are classified according to enzyme or transporter deficiency and organ distribution (Laforêt et al 2011). The hepatic GSDs are inborn errors of glycogen synthesis or breakdown clinically characterized by hepatomegaly, failure to thrive and fasting intolerance, biochemically associated with hypoglycaemia. Both endogenous glucose production (EGP) and metabolic clearance rate of glucose determine blood glucose concentration during fasting. Impairment of EGP may be caused by decreased gluconeogenesis (GNG), decreased glycogenolysis or a combination. Instead of discussing all hepatic GSD types, this review focuses on lipid metabolism and monitoring of dietary management in GSD type I and GSD type III. These GSD types display important differences in both pathophysiology and effects on lipid metabolism. During follow-up, monitoring and prevention from significant long-term morbidity is the rule in GSD I and GSD III, whereas to date, the remaining subtypes (except GSD IV) are generally considered disorders without morbidity in adulthood.

GSD I is caused by a deficiency of the glucose-6-phosphatase (G6Pase) complex (Bali et al 1993). G6Pase is expressed in liver, kidney and intestine and plays a central role in both glycogenolysis and GNG. This explains the severe short-term fasting intolerance and hypoglycemia compared to patients with other hepatic types of GSD. Besides severe nonketotic hypoglycemia, metabolic decompensation is characterized by secondary biochemical abnormalities (hyperlactacidaemia, hyperuricaemia and hyperlipidaemia). Untreated patients classically displayed protruding abdomen (hepatomegaly due to storage of glycogen and fat), short stature, truncal obesity, a rounded doll face, wasted muscles and bleeding tendency. Two major subtypes of GSD I are recognized. GSD type Ia (OMIM #232200) is caused by deficient activity of the catalytic unit of G6Pase, whereas GSD Ib (OMIM #232220) is caused by defect of the putative transporter. In addition to the classical phenotype, GSD Ib patients display recurrent bacterial infections, inflammatory bowel disease and thyroid auto-immunity, associated with neutropenia and neutrophil dysfunction.

The remaining hepatic GSD types (0, III, VI, IX and XI) are associated with fasting ketotic hypoglycemia and considered relatively mild compared to GSD I because GNG is intact (Derks and Smit 2014). In these types of GSD, ketone body (KB) concentrations reflect increased mitochondrial fatty acid oxidation (mFAO), which is proceeded by activation of GNG and secondary endogenous proteolysis from muscle tissue. GSD III (OMIM # 232400) is caused by a deficiency of the debranching enzyme and phenotypically classified as either type IIIa or type IIIb (Dagli et al 1993). GSD IIIa patients (±85 %) display symptoms and signs due to the enzyme deficiency in liver, skeletal muscle and heart, whereas the remaining patients with GSD IIIb (±15 %) have only liver-related phenotypes.

Dietary management in hepatic GSD is traditionally based on the provision of exogenous carbohydrates to compensate defective EGP, to achieve normoglycemia and to correct the secondary metabolic effects as much as possible. In infant GSD I patients, normoglycemia is maintained by frequent (every 1.5–3 h) lactose free formula feeds enriched with maltodextrin. In 1974 continuous nocturnal gastric drip-feeding (CNGDF) via a nasogastric tube was introduced. It was demonstrated that normoglycemia could be maintained (Burr et al 1974; Greene et al 1976), secondary metabolic alterations could be corrected and patients and parents were able to sleep throughout the entire night. In 1984 uncooked cornstarch (UCCS) was described for the first time in GSD patients and developed as either a good alternative or additional strategy to maintain metabolic control (Chen et al 1984; Smit et al 1984). Although UCCS may be introduced at 6 months of age, the tolerance may be reduced as a consequence of lower pancreatic amylase activity until 1 year of age (Hayde and Widhalm 1990). Both CNGDF and UCCS have advantages and disadvantages and differ in many ways, like the optimal age of introduction, duration of normoglycemia, (no) need to wake up during the night, caloric content, glycemic index, risk of technical failure with subsequent metabolic risks, difficulty for parents and/or patients, invasiveness and costs (Derks et al 2013). Dietary management in GSD III patients follows similar principles of exogenous carbohydrates, but the additional role of increasing dietary protein is recognized.

After dietary treatment became available, the phenotype of GSD patients changed from mortality to morbidity and the focus of attention moved towards (prevention of) long-term complications (Moses 2002). For GSD I patients, these complications mainly involve the liver (adenomas and their risks of bleeding and secondary conformation to hepatocellular carcinoma), kidney (glomerular and tubular dysfunction, developing into renal insufficiency), intestine (GSD related enteropathy), growth (end length, body mass index (BMI)), anemia, joints (gout) and bones (osteopenia and osteoporosis) (Bali et al 1993). For GSD III patients, the liver (fibrosis, cirrhosis and hepatocellular carcinoma), muscle (myopathy), heart (cardiomyopathy), growth and bones deserve special attention (Dagli et al 1993).

## Monitoring of plasma lipids in hepatic GSD

In GSD I patients, triglyceride concentration are considered the most useful parameter for chronic metabolic control. Like blood glucose concentrations, lactate concentrations fluctuate rapidly, liver enzymes are only slightly increased before initiation of dietary management and rapidly normalize, and uric acid concentrations can be influenced by allopurinol treatment. In our personal clinical experience with GSD Ia patients, increasing triglyceride concentrations can reflect decreased metabolic control, for instance when young patients outgrow their diet, by decreased compliance, during increased growth in puberty or co-morbidity like hypothyroidism. Based on an expert meeting as part of the European Study on GSD I (ESGSD I), follow-up guidelines for the frequency of routine laboratory investigations (including lipid profiles) are age dependent: age 0–3 years every 2 month; 3–20 years every 3 months; adults every 6 months (Rake et al 2002b).

Cross-sectional cohort data on serum lipid concentrations in GSD1 patients originate from the retrospective multicenter ESGSD I, which included 231 GSD Ia and 57 GSD Ib patients (Rake et al 2002a). Hypercholesterolemia and triglyceridemia were more common and more severe in GSD Ia patients compared to GSD Ib patients. In adult GSD Ia (*n* = 40) and GSD Ib (*n* = 4) patients hypercholesterolemia was noted in 43 % versus 25 %, whereas 98 % versus 75 % displayed hypertriglyceridemia. In this cohort, complications associated with hyperlipidaemia were rare; pancreatitis was reported in three patients and cholelithiasis in two patients. In the management guideline paper originating from 2002, serum triglyceride concentrations <6.0 mmol/L was included as a biomedical target (Rake et al 2002b).

In the ESGSD I the overall prevalence of liver adenomas was 16 % and increased with age (Rake et al 2002a). More recently, Wang and co-workers retrospectively characterized the natural history and factors related to liver adenoma development in GSD1a patients (Wang et al 2011). Patients were stratified in two groups, i.e. patients with mean 5-year triglyceride concentrations ≤ 500 mg/dL (corresponding with 5.6 mmol/L) or > 500 mg/dL, based on the consensus panel discussion at the 2010 Association for Glycogen Storage Disease Conference. The authors reported significantly increased adenoma progression in the latter group. By demonstrating the association between a biomarker (i.e. increased triglyceride concentrations) and outcome (i.e. increased prevalence of liver adenomas), the authors added a new dimension.

In general, hyperlipidemia disappears with age and is milder in the ketotic types of GSD, like GSD III. Bernier et al studied the lipid profiles in 44 GSD III patients ranging from 6 months to 30 years of age (Bernier et al 2008). They demonstrated that the overall prevalences of hypercholesterolemia (31 %) and hypertriglyceridemia (67 %) are lower than in GSD I patients. Hypertriglyceridemia significantly decreased with increasing age in GSD III patients, the median slope was −0.11 mmol/L per year.

## Pathophysiology of lipid metabolism in hepatic GSD

From the point of pathophysiology, several important factors contribute to the difference between GSD I and GSD III.

The fasted state in GSD I is characterized by intracellular accumulation of glucose-6-phosphate with secondary shunting to glycolysis and pentosephosphate route. Glycolysis increases acetyl-CoA production and thereby lipogenesis and malonyl-CoA, the latter inhibiting carnitine palmitoyltransferase I (CPT I), the rate-controlling step of mFAO. Pharmacological inhibition of the glucose 6–phosphate transporter by S4048 induces hypoglycemia in wild type mice, increased hepatic glycogen and triglyceride content, and a markedly enhanced hepatic lipogenic gene expression (van Dijk et al 2001). Human stable isotope studies revealed that GSD Ia is associated with increased rates of de novo lipogenesis and cholesterogenesis (Bandsma et al 2008). After administration of S4048 to several knock-out mice models, the carbohydrate-response-element-binding protein (but not the sterol-regulatory-element-binding protein 1c or liver X receptor α) was identified as an important molecular link mediating the induction of hepatic lipogenic gene expression in GSD I, hence a potential target for pharmacological intervention in human patients (Grefhorst et al 2010). Several metabolites of glycolysis (like glucose-6-phosphate and fructose-2,6-bisphosphate) and the pentosephosphate route (xylulose-5-phosphate) act as activators of the carbohydrate-response-element-binding protein, illustrating the complex interactions between intermediate metabolites and transcription factors (for a recent review, see Oosterveer and Schoonjans 2014).

Experimental data from stable isotope studies demonstrate that EGP is age dependent and decreases relatively with body weight (Bier et al 1977) and age (Huidekoper et al 2014). Because in GSD I patients both glycogenolysis and GNG are impaired, defective EGP and fasting intolerance are more pronounced and lifelong, compared to the ketotic GSD types like GSD III. In the latter group, hyperlipidemia is caused by increased fatty acid flux from adipose tissue to the liver, as an alternative source of energy by mFAO. The decreasing triglyceride concentrations with increasing age goes in parallel with decreasing EGP, reflecting improved fasting tolerance in ageing GSD III patients.

## The role of dietary carbohydrate, fat and protein

First reports about dietary energy percentages of macronutrients in GSD already originate from over four decades ago. Kelsch and Oliver demonstrated that frequent high-carbohydrate feeds improved most metabolic abnormalities in a GSD I patient (Kelsch and Oliver 1969). Fernandes and Pikaar recommended 50–70 and 40–50 % carbohydrates, 15–35 and 30–40 % fat and 15 and 20 % protein, in patients with GSD I and ketotic subtypes, respectively (Fernandes and Pikaar 1969). Fernandes also studied the effects of different disaccharides on blood lactate concentrations and these studies are the experimental base of dietary restrictions of fructose, lactose and saccharose (Fernandes 1974).

Since then, between treatment centers worldwide, major differences have arisen in the practical effectuation of dietary management in GSD (type I). Examples include the type of nocturnal management (UCCS versus CNGDF), the timing of the introduction of UCCS (at which age?), the methods by which exogenous carbohydrates are given and the extent of dietary restrictions of fructose, lactose and saccharose. Recent data from a meta-analysis of three studies suggest that UCCS was associated with significantly higher plasma cholesterol concentrations compared to CNGDF (Shah and O’Dell 2013). There was no significant difference in plasma triglyceride concentrations between these treatments. The difference in lipid profiles between UCCS and heat-treated modified starch has not been systematically studied. In general, these examples emphasize the need of international detailed (dietary) data collection and practical management guidelines appreciating that dietary management in hepatic GSD needs to be individually tailored.

**Fig. 1:**
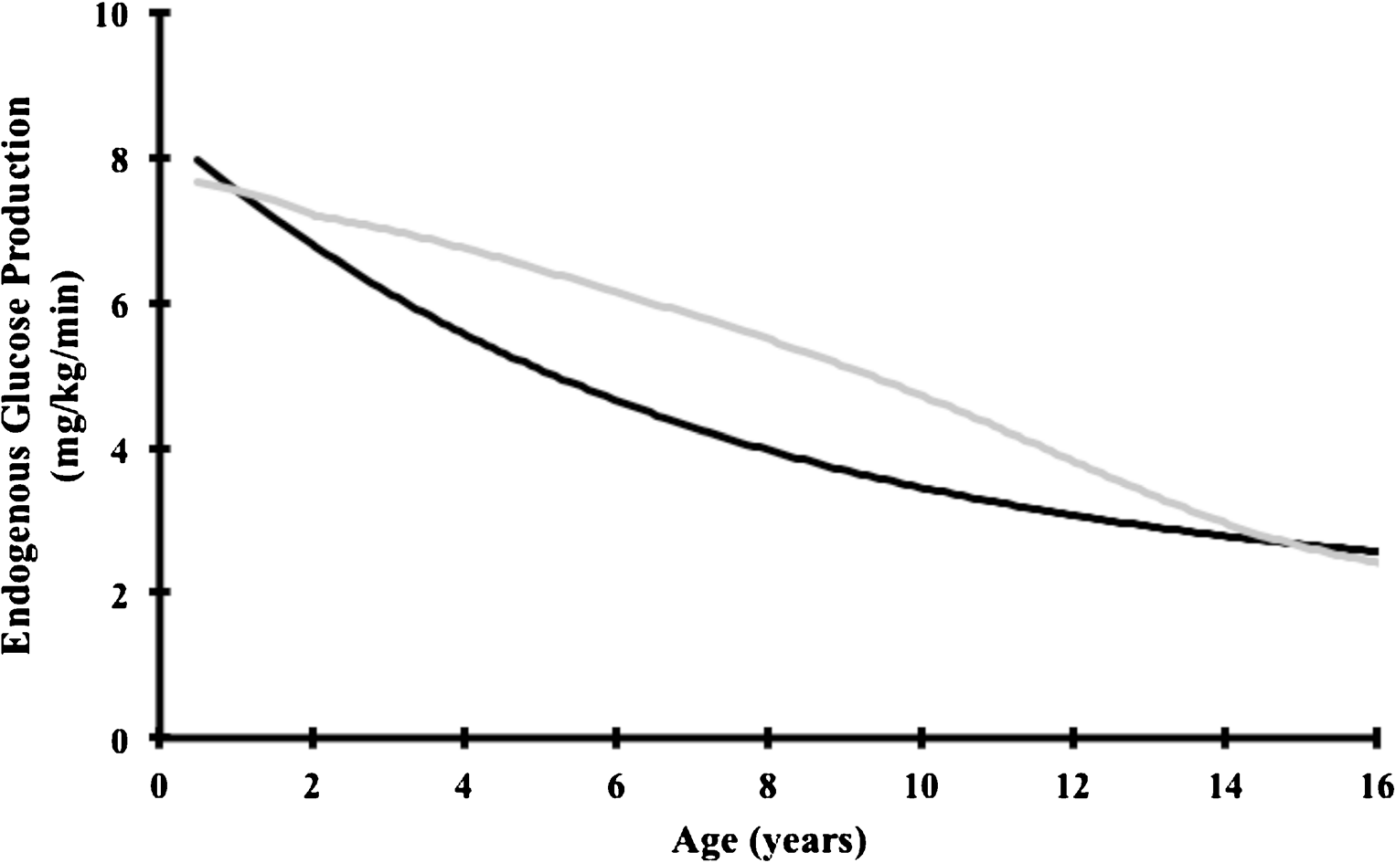
Two regression models to calculate endogenous glucose production, based on body weight (*grey*, by Bier et al 1977) and age (*black*, by Huidekoper et al 2014). The *grey line* represents the EGP regression line according to Bier. Reference values for body weight in boys were retrieved from the World Health Organization and the Centers for Disease Control and Prevention, below 2 years and after 2 years of age, respectively. EGP (expressed in mg/min) is calculated as: 0.0014*x* ^3^–0.214*x* ^2^ + 10.411*x*–9.084, with [x] representing body weight in kg. The *black line* represents EGP regression line according to Huidekoper. EGP (expressed in mg/kg/min) is calculated as: 6 : 50 × 2.72^− 0.145 × *Z*^ + 1.93, with [z] representing age in years

Next to the recommendations about dietary energy *percentages* of macronutrients, data from stable isotope studies on EGP in humans are helpful to estimate the *absolute* carbohydrate requirements. Taking into account, that the theoretical EGP decreases with age, the exogenous glucose requirements for GSD I patients can be estimated with regression formulas derived from these tracer studies. The historical studies by Bier and co-workers have generated a regression formula using body weight as a variable; recently Huidekoper and co-workers generated a regression formula based on age (Fig. 1). Compared to the latter method, the historical method may overestimate carbohydrate requirements because it obviously does not correct for overweight/obesity. At some time points in childhood, the calculation based on age is 25–30 % lower compared to the other method. In addition, both methods share another risk of overtreatment because the tracer studies have been performed in healthy overnight fasted control subjects, a situation different from the perturbed glucose homeostasis in GSD I patients. Experimental data in GSD Ia patients indicate that in vivo EGP is not completely absent and may reach ~60 % of normal (Huidekoper et al 2010). The mechanism is not understood, theoretically, residual in vivo glucose-6-phosphatase-α activity next to alternative glycogenolysis by (muscle) glucose-6-phosphatase-β and/or the α-glucosidase pathway may contribute to whole body EGP in GSD Ia patients.

**Fig. 2:**
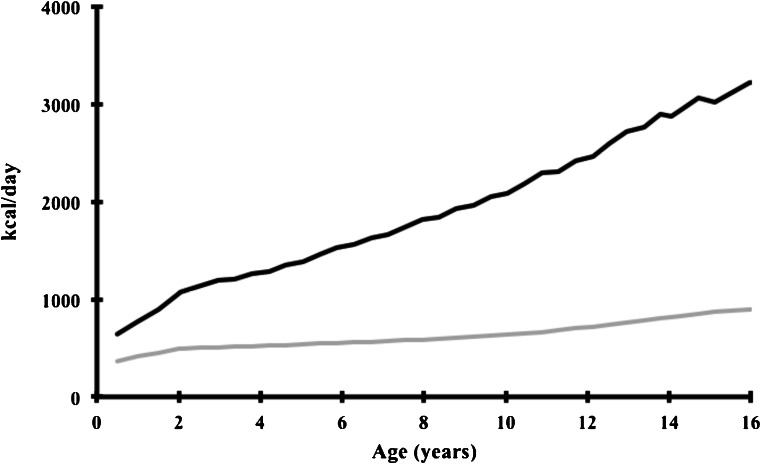
Daily total energy expenditure (*black*) and endogenous glucose production calculated by age (*grey*), both expressed in kcal/day, according to age. Reference values for total energy expenditure (expressed as kcal/kg/day) in boys were retrieved from the Joint FAO/WHO/UNU Expert Consultation on Human Energy Requirements. Reference values for body weight in boys were retrieved from the World Health Organization and the Centers for Disease Control and Prevention, below 2 years and after 2 years of age, respectively. EGP was calculated according to Huidekoper

Figure 2 presents total energy expenditure and EGP in childhood, both expressed in kcal/day. The graph illustrates that the caloric intake by replacement of EGP declines from 60 to 30 % in the first 3 years of life. This may indicate that especially in (early) childhood, dietary management is characterized by a delicate balance between *under*treatment (with hypoglycemias, poor metabolic control and risk of long-term complications) and *overt*reatment (with the risk of rebound hypoglycemias, peripheral body fat storage and obesity). In the European Study on GSD I, about 25 % of the GSD Ia and 33 % of the GSD Ib patients under 20 years of age display BMI above the 90th percentile, interestingly highest prevalence was observed in young patients between 2 and 5 years of age.

From a practical point of view, increasing one macronutrient (carbohydrates) without affecting the second (protein) inevitably lowers fat intake. Given the carbohydrate deficiency and hyperlipidemia in GSD I and GSD III, increasing dietary fat is somewhat counterintuitive. However, there are two positive reports about high-fat diets in GSD III patients. The combination of synthetic ketone bodies, 2:1 ketogenic diet and high-protein was associated with reversal of hypertrophic cardiomyopathy in a 2-months-old GSD IIIa patient (Valayannopoulos et al 2011). In two 7- and 5-year-old siblings, a high-carbohydrate diet was isocalorically replaced by a high fat (60 %) and high protein (25 %, which remained fairly constant), low-carbohydrate (15 %) diet, associated with reversal of hypertrophic cardiomyopathy (Brambilla et al 2014).

Several case reports describe the effects of medium chain triglycerides (MCT) in GSD I and III (Fernandes and Pikaar 1969; Cuttino et al 1970; Nagasaka et al 2007; Hanou et al 2008; Burns et al 2009; Das et al 2010; Bernstein et al 2010; El-Gharbawy et al 2014). In summary, laboratory parameters of metabolic control (triglyceride concentrations in GSD I; transaminases and CK in GSD III) decrease after MCT-supplementation, demonstrating that hyperlipidemia is carbohydrate-induced in GSD I. However, an overall interpretation with conclusions and management advise based on these observations is complicated by differences in age, GSD type and severity (type of mutations) of the disorder, relative and absolute quantities of macronutrients, MCT-sources, indications for MCT-treatment, outcome-parameters, the absence of long-term clinically relevant follow-up data and the possibility of selection bias, unfavourable cases not being reported.

There are no experimental data to substantiate whether long-term dietary MCT may cause liver adenoma and hepatocellular carcinoma. The development of hepatic tumors is enhanced by a high fat enriched diet in liver-specific GSD I deficient mice (Rajas et al 2014), but it is not mentioned whether this depends on the type of fat, i.e. MCT versus long-chain triglycerides. In GSD I *cytoplasmatic* Acetyl-CoA accumulation and subsequent malonyl-CoA formation functionally inhibit mFAO at the level of CPT I. MCT are absorbed via the portal vein and the released free fatty acids enter mFAO without the carnitine shuttle. The beta-oxidation generates *intramitochondrial* Acetyl-CoA for direct ketogenesis. It can be hypothesized that increased ketogenesis by MCT decreases the metabolic clearance rate of glucose in GSD I patients, although the exact molecular mechanism is still unknown.

MCT can be supplied by naturally occurring dietary products, medical dietary supplements, or pharmaceutically (triheptanoin). For all methods, the potential risk of glycerol overload towards impaired GNG in GSD I patients is unknown. Triheptanoin is a medium-chain triglyceride of 7-carbon fatty acids observationally studied in patients with long-chain mitochondrial fatty acid oxidation disorders. Besides the ease of use, the anapleurotic effect of triheptanoin GSD Ia could be beneficial.

The role of dietary protein in GSD I has not systematically been studied and has recently been reviewed for GSD III (Derks and Smit 2014).

## Discussion and future directions

In summary, hepatic GSD underscores the intimate relations between carbohydrate and lipid metabolism. Hyperlipidemia is most pronounced and often permanent in GSD I, in whom the intrahepatic degree of metabolic control is reflected by blood lipid concentrations. Hypertriglyceridemia is associated with pancreatitis and liver adenomas in GSD I patients. In ketotic GSD patients, hyperlipidemia reflects lipolysis from extrahepatic sources, associated with the age-dependent fasting intolerance.

Like often in rare diseases, medical emergencies in individual patients initiated (dietary) interventions and generated new hypotheses. Future experimental animal studies and well-designed collaborative dietary intervention studies are necessary to improve our understanding about manipulations in macronutrients, like the restrictions of simple sugars, traditional versus heat-treated modified starch, the role of protein, MCT-treatment, triheptanooin and the ketogenic diet in GSD type III. International patient registries will be necessary to study outcome and the effects of (elements of) dietary management.

In the next decades, dietary management of GSD patients will be individualized more than ever, integrating personal metabolic and genetic data to optimize metabolic control. Continuous glucose monitoring systems are used in GSD patients in the real life situation to improve dietary management and thereby parameters of metabolic control, including the lipid profile (Hershkovitz et al 2001; White and Jones 2011; Kasapkara et al 2014). Nowadays, the most modern (and expensive) devices alarm at low glucose levels, significantly improving safety. Near infra red spectroscopy is a promising technique for non-invasive measurement of blood glucose (Xue et al 2014) and the integration of this technique in the glucose monitoring systems would add new dimensions to the safety and acceptability of dietary management of GSD patients.

The definition of the metabolic syndrome and their criteria has been revised several times, leading to multiple definitions in current use. However, the general concept is that patients have a combination of conditions, among which are insulin resistance or hyperglycemia, hyperlipidemia, hypertension and obesity. Regardless of which definition, GSD I patients meet some of the criteria applied. Currently there are conflicting experimental data (Ubels et al 2002; Bernier et al 2009) and no convincing epidemiological data substantiating strong associations between metabolic syndrome, type 2 diabetes and GSD. Close clinical monitoring for cardiovascular diseases, chronic kidney disease and diabetes in ageing GSD patients (regardless what subtype) seems to be justified, because these disorders share many metabolic pathways (Rajas et al 2013).

## Abbreviations

BMI: Body mass index
CNGDF: Continuous nocturnal gastric drip-feeding
CPT I: Carnitine palmitoyltransferase I
EGP: Endogenous glucose production
ESGSD I: European Study on GSD I
G6Pase: Glucose-6-phosphatase
GNG: Gluconeogenesis
GSD: Glycogen storage disease
MCT: Medium-chain triglycerides
mFAO: Mitochondrial fatty acid oxidation
UCCS: Uncooked cornstarch
